# Ethical Governance of Large Language Models in Health Care: Trust, Responsibility, and Equity in Routine Use

**DOI:** 10.2196/93470

**Published:** 2026-06-10

**Authors:** Xiongwen Yang, Chuan Xu

**Affiliations:** 1 Department of Thoracic Surgery, Guizhou Provincial People's Hospital, 83 Zhongshan East Road, Guiyang, Guizhou, 550002, China, 86 18620726507, 86 18620726507; 2 NHC Key Laboratory of Pulmonary Immunological Diseases, Guizhou Provincial People's Hospital, Guiyang, Guizhou, China

**Keywords:** large language models, medical ethics, ethical governance, health care communication, technology adoption, equity and trust, responsibility

## Abstract

Large language models (LLMs) are becoming increasingly embedded in routine health care communication, raising ethical challenges that extend beyond model performance alone. This Viewpoint argues that ethical risks in LLM-enabled health care emerge through patterns of reliance, institutional embedding, and governance during real-world use. Using “adoption-phase ethics” as an analytic lens, this paper examines 3 interrelated dimensions of ethical risk. First, trust in LLM-enabled health care is shaped not only by technical accuracy, but also by institutional and relational conditions surrounding its use. Second, responsibility may become distributed and ambiguous when LLM-mediated information influences clinical communication and decision-making. Third, equity concerns arise from unequal capacities to interpret, contest, and benefit from LLM-generated information. We argue that ethical governance of LLMs in health care requires continuous, system-level oversight that extends beyond model evaluation alone, including clear accountability structures, role-sensitive implementation, and equity-oriented governance practices. By reframing ethical analysis around routine integration rather than technical performance alone, this Viewpoint aims to support more responsible and sustainable use of LLMs in health care.

## Introduction

Large language models (LLMs) have rapidly evolved from experimental research tools to increasingly visible participants in health care information ecosystems. Early investigations have largely focused on whether these systems can perform clinically relevant tasks—such as summarizing medical documentation, clarifying technical terminology, or supporting elements of decision-making—with acceptable levels of accuracy and efficiency [1-3]. Such performance-oriented research has been essential in establishing technical feasibility and translational promise.

Recent advances in medical artificial intelligence (AI) have also explored techniques such as multicenter knowledge transfer, multimodal learning, and domain adaptation to improve model robustness and generalizability [4-7]. While these approaches primarily address technical performance, they further highlight the importance of considering how such systems are deployed, interpreted, and governed in real-world contexts. As LLMs begin to enter routine clinical and informational contexts, however, additional ethical questions emerge. These questions cannot be addressed solely by whether outputs meet predefined benchmarks under controlled evaluation. Rather, they concern how ethical responsibilities, patterns of reliance, and expectations of accountability may shift when LLMs are integrated into everyday care practices [8-11].

In this Viewpoint, we use the term “adoption-phase ethics” as an analytic lens for examining how ethical risks emerge during the sustained integration of LLMs into routine health care practice. Rather than focusing primarily on predeployment evaluation or technical adoption alone, this perspective emphasizes how patterns of reliance, institutional embedding, and governance arrangements shape ethical outcomes during real-world use.

During experimental evaluation, ethical attention often centers on technical properties such as performance, validation, bias mitigation, and explainability [12]. These concerns remain crucial. Improvements in model accuracy and robustness can meaningfully reduce certain risks. Routine integration raises ethical questions that extend beyond technical performance alone. As LLM-mediated information becomes embedded within clinical communication and patient interpretation practices, ethical concerns increasingly involve how trust is formed, how responsibility is distributed, and how individuals interpret uncertainty within institutional contexts [10,11,13,14]. These dynamics are particularly significant in health care, where patient vulnerability, professional accountability, and asymmetries of expertise shape reliance on AI-mediated information [11,13,15,16]. Governance conditions, including institutional accountability and uncertainty communication, therefore become central to ethical integration during routine use.

These considerations invite a reframing of AI ethics in medicine. At its core, this Viewpoint argues that the central ethical challenge of LLM integration in health care lies not simply in model performance, but in how patterns of reliance become institutionalized during routine use. Much prior work has emphasized predeployment evaluation, such as performance validation, bias assessment, and transparency requirements. While these remain foundational, ethical challenges in practice do not replace model-level concerns but interact with them in complex ways. Model-level properties, including accuracy, bias, and uncertainty, remain ethically significant. However, ethical risks during routine use are shaped not only by these technical characteristics, but also by how LLMs are embedded within institutional workflows, communication practices, and governance structures. Ethical analysis must therefore extend beyond technical evaluation toward questions of accountability, reliance, and equitable capacity to benefit during sustained use [17,18].

In this Viewpoint, adoption is understood not merely as technical deployment, but as the stabilization of LLM use within everyday clinical and informational practices. Drawing on normative ethical analysis informed by existing empirical discussions, we examine 3 interrelated domains—trust, responsibility, and equity—to clarify how routine LLM use may (1) reshape institutional and interpersonal trust, (2) create responsibility gaps, and (3) amplify disparities in interpretive and practical capacity.

This Viewpoint contributes to existing discussions by reframing ethical analysis from a predominantly model-centric perspective toward one that is governance-oriented and grounded in routine use. It conceptualizes “adoption-phase ethics” as an analytic lens for understanding how patterns of reliance, institutional embedding, and differential capacity shape ethical risk during sustained LLM integration.

This perspective differs from many existing AI ethics frameworks, which primarily emphasize general principles such as fairness, transparency, and accountability at the model or system design level [19-21]. While existing AI ethics frameworks provide important normative guidance, they often emphasize ethical principles as properties to be ensured prior to deployment. In contrast, this perspective focuses on how ethical risks evolve through patterns of reliance, institutional embedding, and governance during routine use [19-22].

This Viewpoint focuses on LLM applications in communication support and non-autonomous decision contexts, rather than fully autonomous diagnostic systems. It aims to examine how ethical risks emerge during routine integration and to propose a governance-oriented framework grounded in trust, responsibility, and equity.

This analysis is structured around 3 key messages. First, trust in LLM-enabled health care is shaped not only by model performance, but also by institutional endorsement and governance structures. Second, responsibility becomes distributed across clinicians, institutions, and system designers, creating potential ambiguity in accountability. Third, equity concerns arise from differences in users’ capacity to interpret and benefit from AI-mediated information.

This Viewpoint is intended for clinicians, health system leaders, and policymakers engaged in the governance and implementation of LLM-enabled health care systems.

## Conceptual Approach

As shown in Table S1 in Multimedia Appendix 1, recent empirical studies consistently highlight that ethical challenges in LLM use extend beyond model performance and are shaped by patterns of reliance, responsibility allocation, and governance conditions in real-world settings.

Conceptually, this analysis is situated at the intersection of AI ethics, health care technology adoption, and sociotechnical systems theory. Drawing on existing empirical and conceptual scholarship, this Viewpoint develops a governance-oriented analytical lens for examining how ethical risks emerge during the routine integration of LLMs into health care. Rather than focusing solely on model performance or adoption outcomes, the analysis emphasizes how patterns of reliance, responsibility allocation, and differential capacity to benefit are shaped through sustained real-world use.

The analysis is structured around 3 interrelated domains: trust, responsibility, and equity. These frequently arise in discussions of health care technology integration. Trust refers to institutional and relational expectations surrounding the reliability and governance of LLM-mediated information. Responsibility concerns how accountability and decision authority are distributed among clinicians, institutions, and system designers. Equity addresses differences in access, interpretive capacity, and practical benefit across user groups. Together, these domains provide a framework for examining how ethical risks become embedded within sociotechnical systems during routine LLM use.

**Figure 1.**
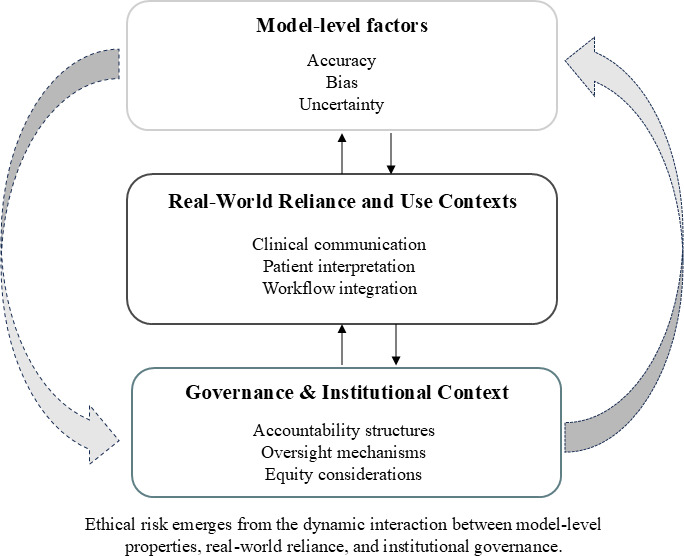
Interaction-based framework of ethical risk in large language model–enabled health care.

The analysis focuses on LLM applications in communication support and nonautonomous decision contexts within health care. Figure 1 illustrates the interaction between model-level properties and system-level governance conditions, emphasizing that ethical risks emerge through their ongoing interplay during routine use rather than through a strictly linear transition between stages.

This figure presents a layered and interaction-based framework for understanding ethical risk during routine LLM integration in health care. Model-level properties (eg, accuracy, bias, and uncertainty), patterns of reliance in clinical practice (eg, communication, interpretation, and workflow integration), and governance conditions (eg, accountability structures, oversight mechanisms, and equity considerations) are conceptualized as interacting dimensions within a sociotechnical system.

Rather than representing a linear progression from model evaluation to implementation, the framework emphasizes that ethical risks emerge dynamically through the institutionalization of reliance during real-world use.

## Ethical Dynamics of Routine LLM Adoption in Health Care: Trust as an Institutional and Relational Phenomenon

As LLMs move from experimental evaluation into routine clinical and informational contexts, questions of trust take on heightened ethical significance [2,23]. While technical performance remains relevant, trust during sustained use cannot be understood solely as confidence in algorithmic accuracy [16,24]. Instead, it is shaped by expectations regarding transparency, accountability, and institutional protection.

In practice, clinicians often evaluate LLM-supported communication not only on perceived output quality but also on whether use is embedded within governed workflows that clarify oversight and professional responsibility. For example, when LLMs generate patient-facing explanations of diagnostic results, clinicians may need to assess not only factual accuracy but also whether uncertainty is appropriately communicated and aligned with clinical judgment. For patients and caregivers, trust may depend on whether LLM-mediated information is intelligible, appropriately contextualized, and clearly distinguished from professional clinical judgment [9,25,26].

Taken together, these dynamics suggest that trust in adoption-phase LLM use is not merely an individual attitude toward technology. Rather, it emerges as a relational and institutional phenomenon, structured by governance signals that communicate who stands behind LLM-mediated information and how harms would be addressed if they occur. Ethical analysis must therefore consider not only whether models are accurate, but also how institutional endorsement and accountability shape patterns of reliance [10,27,28].

## Responsibility and Accountability in Mediated-Information Environments

Routine integration of LLMs into health care communication also raises questions about responsibility allocation. Clinicians remain legally and ethically accountable for patient care, yet LLM-generated summaries, explanations, or drafts may mediate access to information in ways that obscure provenance, limitations, or uncertainty [26,28,29]. This issue is particularly pronounced in health care, where professional responsibility is formally codified, and where reliance on mediated information may have direct implications for patient safety and clinical accountability.

At the same time, patients and caregivers encountering AI-mediated explanations may struggle to distinguish between professional clinical guidance and automated inference. For instance, a patient receiving an LLM-generated explanation of treatment options may interpret the information as authoritative medical advice, even when it lacks formal clinical validation or contextualization. When boundaries are unclear, interpretive burdens can shift onto users who lack equivalent expertise or institutional recourse. This configuration may create the possibility of responsibility gaps—not necessarily because of technical error, but because of structural ambiguity in how accountability is distributed across clinicians, institutions, and system designers [24,27,29].

Importantly, these concerns are not exhausted by improvements in technical performance. Even highly accurate systems may introduce new ambiguities regarding authorship, endorsement, and liability when embedded into routine workflows [28]. Ethical evaluation must therefore address how responsibility is specified, communicated, and governed in contexts where AI mediates clinical information exchange.

## Equity, Capability, and Differential Vulnerability

A further ethical dimension of routine LLM adoption concerns uneven capacity to interpret and benefit from AI-mediated health information. Engagement with digital tools is shaped by differences in digital literacy, educational background, language proficiency, and access to institutional support. These factors influence not only initial uptake but also users’ ability to contextualize outputs, recognize uncertainty, and seek clarification when needed [10,30,31].

In health care settings, these disparities may have heightened ethical significance, as misinterpretation of information can directly affect patient understanding, decision-making, and health outcomes, particularly among vulnerable populations. In certain contexts, LLMs may function as capability amplifiers: they can enhance access and understanding for users equipped to navigate them effectively, while offering more limited or potentially confusing benefits to those with fewer resources. Users with greater digital literacy may be better positioned to contextualize or verify LLM-generated information, whereas others may rely on it more passively, increasing the risk of misunderstanding or misplaced trust. Without deliberate governance attention, patterns of reliance may therefore reproduce or intensify existing inequities [31,32].

Ethical vulnerability is also asymmetrically distributed across user roles. Patients and caregivers may face epistemic vulnerability when interpreting LLM-mediated information without sufficient authority or recourse [28]. Clinicians, by contrast, may face risks of overreliance, cognitive offloading, or erosion of professional judgment under time pressure. These vulnerabilities differ in kind and degree, underscoring that ethical risks associated with routine adoption are not evenly shared [24,26].

## Rethinking AI Ethics Frameworks in Light of Adoption

The dynamics described above invite reconsideration of how AI ethics frameworks are applied to LLM integration in health care. Much prior work in medical AI ethics has emphasized principles such as transparency, explainability, fairness, and bias mitigation—often in the context of predeployment evaluation [7,33,34]. These principles remain foundational. However, routine adoption surfaces ethical challenges that extend beyond model-level properties [17,18,25].

Transparency, for example, is frequently framed as explainability of internal model logic. Yet in practice, users may be more concerned with institutional endorsement, accountability, and recourse mechanisms than with technical architecture. Similarly, fairness is often operationalized through statistical metrics, while disparities in digital literacy, infrastructure, and implementation context may shape who can meaningfully benefit from LLM-mediated information [16,24].

Another limitation of prevailing frameworks lies in their implicit individualization of responsibility. Ethical guidance often presumes that informed users can appropriately interpret and contest AI-generated information. In routine care environments characterized by time pressure and asymmetries in expertise, such assumptions may be unrealistic. Responsibility for ethical use cannot plausibly rest on individual vigilance alone; it must be institutionally structured and explicitly governed [26,29].

Finally, ethical evaluation is sometimes treated as a static, predeployment exercise. Adoption, by contrast, is dynamic. Patterns of reliance evolve, user expectations shift, and organizational norms adapt over time. Ethical oversight must therefore be conceived as an ongoing governance process responsive to emerging vulnerabilities rather than as a one-time compliance checklist [29].

## Governance-Oriented Considerations for Routine Integration

If ethical risks become more pronounced during sustained adoption, governance responses must likewise shift in emphasis [18,27]. The patterns summarized in Table S1 in Multimedia Appendix 1 further demonstrate that governance challenges arise not only from model limitations, but from the ways in which LLMs are embedded within clinical workflows and relied upon in practice.

First, trust in LLM-supported communication should be institutionally anchored. At an operational level, governance mechanisms should be embedded within clinical workflows through structured human-in-the-loop review, audit trails documenting LLM-generated outputs and clinician modifications, and clear escalation pathways for uncertain, contested, or potentially harmful outputs. Health care organizations should also clarify how LLM outputs are validated, monitored, and integrated into communication and decision-making processes.

Second, governance should be role-sensitive. Clinicians and patients engage with LLMs under different conditions of authority, expertise, and exposure to harm. Ethical design should therefore support calibrated reliance and auditability for clinicians while ensuring intelligibility, uncertainty signaling, and accessible recourse for patients and caregivers. Examples of accessible recourse include clearly defined complaint or feedback pathways, mechanisms for correcting or retracting erroneous AI-generated information, and the availability of human support to review and clarify contested outputs. Such mechanisms should be clearly assigned to responsible actors (eg, clinical teams and institutional oversight committees) and embedded in patient-facing communication channels to ensure that users can request clarification, correction, or human review in a timely manner.

Third, equity requires proactive attention. Literacy-sensitive interfaces, adaptive explanatory strategies, and targeted support for underserved populations should be regarded as central components of ethically robust adoption. Without such measures, LLM integration risks amplifying disparities under the banner of informational democratization.

Finally, oversight should be continuous. Because adoption is an evolving process, health care systems must establish mechanisms for monitoring, feedback, and iterative revision. An ethics of adoption thus centers on institutional responsibility, inclusivity, and sustained governance rather than on technical safeguards alone. This may involve periodic auditing of system use, monitoring patterns of reliance and error, and incorporating user feedback into governance updates and system redesign.

## Conclusion

LLMs are becoming increasingly embedded within everyday health care communication, reshaping how medical information is accessed, interpreted, and relied upon. In this Viewpoint, we argue that ethical challenges in LLM-enabled health care are not driven by model performance alone, but emerge through patterns of reliance, institutional embedding, and governance during routine use.

This analysis reframes ethical evaluation from a predominantly model-centric perspective toward a governance-oriented perspective grounded in real-world integration. By conceptualizing “adoption-phase ethics” as an analytic lens, this paper has highlighted how trust, responsibility, and equity interact as dynamic dimensions of ethical risk during sustained LLM use.

Several limitations should be acknowledged. This analysis is conceptual and does not provide empirical validation of the proposed framework. In addition, while we draw on emerging empirical studies to inform the discussion, the synthesis is not exhaustive and may not capture all implementation contexts.

Future research should examine how governance mechanisms and patterns of reliance shape ethical outcomes in clinical practice, as well as how context-specific governance strategies can be developed and evaluated across different health-care settings.

Ultimately, ethical integration of LLMs in health care will depend not only on technical performance, but also on how institutional governance structures shape reliance, accountability, and equitable capacity to benefit users during routine use.

## Supplementary material

10.2196/93470 Multimedia Appendix 1 Empirical studies informing trust, responsibility, and equity considerations in routine LLM integration in health care.

## Abbreviations

AI: artificial intelligence
LLM: large language model
